# ExtraPEG: A Polyethylene Glycol-Based Method for Enrichment of Extracellular Vesicles

**DOI:** 10.1038/srep23978

**Published:** 2016-04-12

**Authors:** Mark A. Rider, Stephanie N. Hurwitz, David G. Meckes

**Affiliations:** 1 Department of Biomedical Sciences, Florida State University College of Medicine, Tallahassee, 32306, FL, USA.

## Abstract

Initially thought to be a means for cells to eliminate waste, secreted extracellular vesicles, known as exosomes, are now understood to mediate numerous healthy and pathological processes. Though abundant in biological fluids, purifying exosomes has been challenging because their biophysical properties overlap with other secreted cell products. Easy-to-use commercial kits for harvesting exosomes are now widely used, but the relative low-purity and high-cost of the preparations restricts their utility. Here we describe a method for purifying exosomes and other extracellular vesicles by adapting methods for isolating viruses using polyethylene glycol. This technique, called ExtraPEG, enriches exosomes from large volumes of media rapidly and inexpensively using low-speed centrifugation, followed by a single small-volume ultracentrifugation purification step. Total protein and RNA harvested from vesicles is sufficient in quantity and quality for proteomics and sequencing analyses, demonstrating the utility of this method for biomarker discovery and diagnostics. Additionally, confocal microscopy studies suggest that the biological activity of vesicles is not impaired. The ExtraPEG method can be easily adapted to enrich for different vesicle populations, or as an efficient precursor to subsequent purification techniques, providing a means to harvest exosomes from many different biological fluids and for a wide variety of purposes.

Exosomes are virus-sized membranous sacs released from cells^1^,^2^. They contain protein and nucleic acid cargo that mediate intercellular communication^3^,^4^,^5^,^6^. Exosomes are present in diverse biological fluids and contain molecular signatures of their progenitor cells^6^. Additionally, the discovery that exosomes participate in pathological conditions has generated tremendous interest in uncovering their functions, and efforts to discover biomarkers in exosomes for diagnostic or prognostic purposes have intensified^7^,^8^,^9^,^10^,^11^,^12^,^13^,^14^. Exosomes are distinguished from other extracellular vesicles by size, content, and location of their biogenesis^2^. Whereas other extracellular vesicles, like microvesicles, bud off from the cell surface, exosomes are generally regarded as vesicles that originate at internal endosomal membranes of multivesicular bodies (MVB), and are released into the extracellular milieu following fusion of the MVB with the plasma membrane^15^.

The most commonly used exosome enrichment procedure is differential centrifugation (DC): a series of centrifugation steps of increasing centrifugal force (typically up to 100,000 g and greater) used to eliminate progressively smaller unwanted debris and larger subpopulations of vesicles^16^,^17^. Exosome isolation is complicated by the fact that vesicle subpopulations are not thoroughly defined and may overlap in size and density^3^,^18^. Furthermore, there is ongoing debate about the presence of exosome subpopulations with unique cargo and mechanisms of formation^15^,^19^. This makes achieving truly pure vesicle preparations empirically impossible. Adding to this problem is the great expense and handling time required for enrichment and purification, especially for proteomics experiments that require concentrating large volumes of media for extracting vesicle protein. Recently, user-friendly but expensive commercial precipitation reagents like ExoQuick^TM^ from Systems Biosciences, and Total Exosome Isolation^TM^ from Life Technologies have become popular. Despite widespread use of these reagents, very little has been reported about the quality and quantity of the exosomes harvested compared to other methods.

ExoQuick (patent number: US20130337440 A1) and Total Exosome Isolation (TEI; US20130273544 A1) reagents contain volume-excluding polymers (e.g.: polyethylene glycol, dextrans, or polyvinyls). However, simple solutions of polyethylene glycol (PEG) have been used for over fifty years to concentrate and purify viruses^20^. Because exosomes and virus particles have similar biophysical properties, we hypothesized that a PEG-based method used for virus isolation could be modified to enrich and purify exosomes, providing an inexpensive and efficient alternative to commercially available products and DC. We developed and optimized a protocol for exosome enrichment based on Mahy and Kangro’s compendium on the use of PEG for virus enrichment^21^. To assess our adapted protocol, we first attempted to enrich vesicles from healthy cell cultures. Then, we aimed to evaluate the practical utility of this method by comparing it to the most commonly used methods for enriching vesicles and exosomes. Finally, we tested the versatility of this method by investigating the biological activity of enriched vesicles, the ability to collect from them intact protein and RNA, and by evaluating vesicle yields from a diversity of biological fluids. Overall, we sought a rapid, inexpensive, and adaptable means of harvesting large amounts of exosomes without sacrificing the purity of the preparation.

## Results

### Polyethylene glycol treatment of cell culture medium enriches exosomes

**Figure 1:**
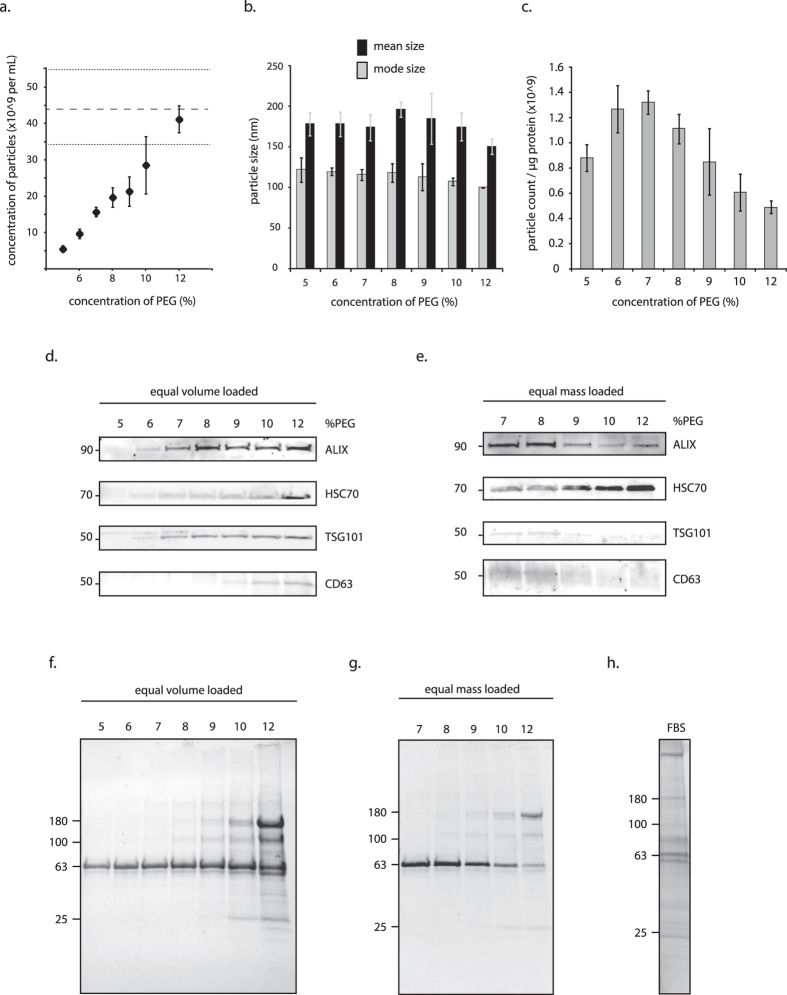
Polyethylene glycol enriches vesicles containing exosome proteins. (**a**) Nanoparticle tracking analysis identified particles in all PEG treatments. The greatest number of particles were recovered using 12% PEG (dashed line represents baseline particle count: particles harvested from conditioned medium assessed by NTA prior to PEG treatment; bounded by one standard deviation). (**b**) No differences in particle size between groups were detected. (**c**) Purity was plotted as a ratio of particles recovered to micrograms of protein in sample. Sample purity peaked when PEG concentrations of 6 to 8% were used. (**d,e**) Lysates were evaluated for exosome protein. Equal-volume (35uL) or equal-mass (8 μg) gel-loading was used to evaluate exosome recovery and purity, respectively. (**f,g**) Blots were Ponceau stained to evaluate total protein, and compared to (**h**) fetal bovine serum protein (FBS) alone (protein recovered from PEG-treated, unconditioned culture medium, with 10% FBS). All approximate protein masses are represented in kilodaltons (kDa).

To demonstrate that a simple adaptation of the virus enrichment method would enrich exosomes, PEG solutions were added to culture media at concentrations between 5 and 12% and refrigerated overnight. The following day, samples were centrifuged for 1 hr at maximum speed in a standard tabletop centrifuge (to maximize ease of processing large volumes of media). The resulting pellets were suspended and particles were characterized using a nanoparticle tracker. Regression analysis indicated a strong positive association between the concentration of PEG and particle numbers recovered (*p* < 0.001*, adjusted R-squared* = 0.93; Fig. 1a). All experimental conditions yielded exosome-sized particles, and particle size (mean and mode) did not differ significantly between any of the groups (Fig. 1b). There was, however, a negative association between mode particle size and PEG concentration (*p* < 0.01*, adjusted R-squared* = 0.35). This suggests that higher PEG concentrations foster precipitation of smaller extracellular vesicles. Alternatively, these results could represent increased precipitation of off-target protein aggregates. As serum proteins are common contaminants in vesicle preparations from cell culture, the purity of the preparations was also assessed by calculating the ratio of particle numbers harvested per total protein harvested (particle number in 1 mL medium per microgram quantity of protein in the same volume medium; Fig. 1c)^22^. Purity of the preparations peaked with PEG concentrations of between 6 and 8% (with no statistical difference between those sample groups). At 9% PEG, there was a statistically significant drop in purity from the maximum (*p* = 0.043).

The presence of targeted vesicles was confirmed by western blot analyses probing for the markers of exosomes, TSG101, CD63, HSC70, and ALIX^16^. Each lane of the gel contained an equal volume of lysate from a treatment group (Fig. 1d, *equal volume loaded*). Samples using higher PEG concentration produced greater signal intensity, corroborating the nanoparticle tracking results. The same samples were then reevaluated, but this time an equal microgram quantity of each lysate was loaded into the gel to assess purity. Precipitation of off-target proteins was expected to increase with increasing concentrations of PEG, thus reducing the amount of exosome protein loaded into the gel. As expected, for most markers signal strength peaked in groups treated with 7 to 8% PEG (Fig. 1e, *equal mass loaded*). The pattern of HSC70 detection by western blot differed from the other markers, suggesting that HSC70 may be enriched in smaller vesicle populations or protein complexes that are preferentially isolated using higher PEG concentrations. Finally, qualitative assessment of the proteins was conducted by staining the nitrocellulose membrane blot with Ponceau S solution (Fig. 1f,g). Similar banding patterns appeared in lanes loaded with FBS alone, suggesting serum protein predominated amongst the harvested protein (see also Fig. 1h). As PEG concentration increased, so did the quantity and composition of serum proteins that co-precipitated with vesicle proteins. Consistent with the western analysis, at 7 to 8% fewer unique serum proteins were harvested.

### PEG-based vesicle enrichment is comparable or superior to standard and commercial methods

**Figure 2:**
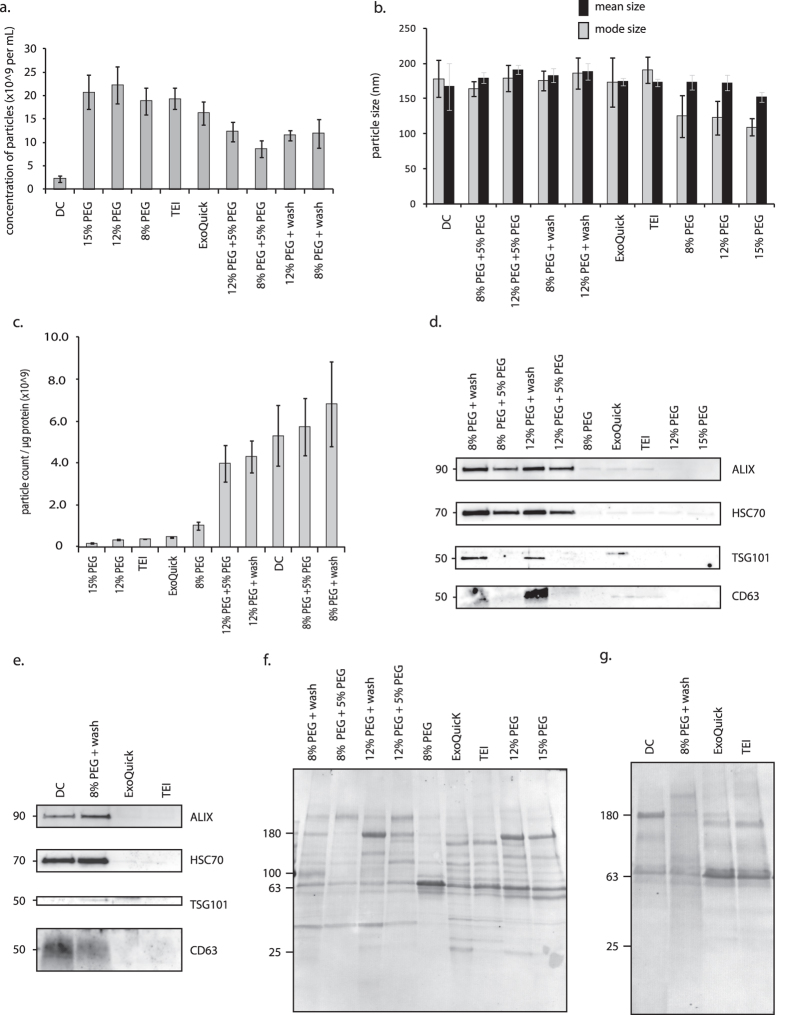
PEG-based vesicle enrichment is comparable or superior to standard harvest methods. (**a**) All harvest methods recovered more particles than differential centrifugation (DC). (**b**) Particle sizes were consistent with the reported size distributions of exosomes. (**c**) The average sample purity of the *8% PEG* + *wash* group was not statistically different from the DC group (*p* > 0.05), but was purer than those of the commercial products (*p* < 0.01). (**d**) Of the PEG-based methods, *8% PEG* + *wash* and *12% PEG* + *wash* produced the strongest evidence of exosomes by western blot. (**e**) Western analyses showed that purity of *8% PEG* + *wash* group was comparable to DC, and superior to commercial methods. (**f,g**) Ponceau staining demonstrated that no method removed all serum protein contaminants, however, the *8% PEG* + *wash* method greatly reduced the most prominent contaminants relative to other treatments. *A non-specific >50 kDa band appeared in the ExoQuick sample lane (**d**). It was subsequently confirmed to not reflect the presence of TSG101 (see Fig. 3). TEI, Total Exosome Isolation (Life Technologies). All approximate protein masses are represented in kilodaltons (kDa).

Having confirmed that the enriched particles contained exosome protein, techniques were explored for improving yield and purity, and these methods were compared to the commercially available ExoQuick and Total Exosome Isolation methods. As the 8% PEG solution yielded high amounts of particles without significantly sacrificing purity (Fig. 1c), this concentration was used as a reference, along with the 12% concentration group that yielded the most particles overall (Fig. 1a). These PEG groups were compared to identical treatments that were further processed by one of two methods: either a secondary PEG treatment, or PBS wash by ultracentrifugation (100,000 g). The PEG methods were also compared to the commercial methods and DC. The commercial and PEG methods yielded more particles compared to DC (Fig. 2a). Particle size was uniform, although notably the PEG methods of higher concentrations of polymer harvested particles with a smaller mode size than the DC group (*p* < 0.05), consistent with observations noted in Fig. 1 (Fig. 2b).

More striking differences were evident with regard to purity: treatments with a final ultracentrifugation wash step (including DC samples), or with a secondary PEG treatment, produced purer particles than samples with PEG treatment alone (Fig. 2c). There was no difference in purity between the most pure outcome (*8% PEG* + *wash*) and the DC method (*p* = 0.344). Methods that employed a secondary PEG treatment recovered fewer exosomes than methods utilizing an ultracentrifugation wash (Fig. 2d). The most pure method (*8% PEG* + *wash*) was then compared to the commercial methods and DC. The *8% PEG* + *wash* group produced preparations comparable in exosome-marker intensities to the DC standard (Fig. 2e). The commercial methods produced less evidence of exosomes, in spite of having collected more particles. This was likely due to the lower purity of the preparations, which resulted in less exosome-specific protein being loaded into the gel. After extended exposure times, ALIX and HSC70 protein bands were evident in TEI and ExoQuick preparations (not shown). Staining showed that the methods differed in unique proteins recovered (Fig. 2f,g). Of note, the *PEG 8%* + *wash* method precipitated a wider distribution of protein sizes without favoring the enrichment of the major contaminating serum proteins, such as the dense 63 kilodalton (kDa) band (Fig. 1h) evident in other preparations. Ponceau staining verified that the commercial reagents indeed successfully precipitated protein, though these isolates contained fewer exosome-specific proteins, evidenced by the western blots.

### Highly pure exosomes can be isolated with polyethylene glycol

**Figure 3:**
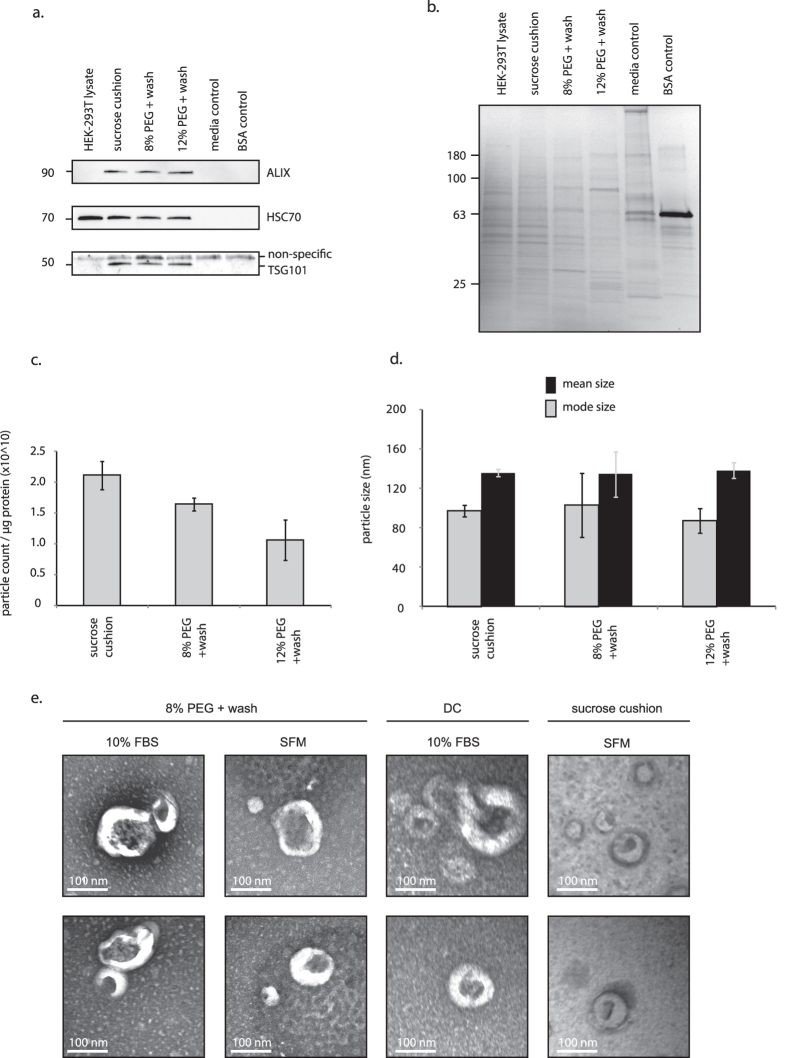
Highly pure extracellular vesicles can be enriched using PEG. (**a**) Western blot was used to assess the purity of samples from conditioned serum-free media (SFM). Abundances of exosome markers in PEG-treated samples were comparable to the sucrose cushion isolate. Probing for TSG101 produced a non-specific band at the 55–60 kDa marker; the non-specific band appeared consistently in samples containing only stock medium with 10% FBS, and in the bovine serum albumin (BSA) negative control. (**b**) Gels were coomassie-stained to compare total protein isolated from sucrose cushion and PEG methods. (**c**) The *8% PEG* + *wash* method produced highly pure samples, comparable to the sucrose cushion isolates (no statistical difference, *p* = 0.169). (**d**) No differences in particle size were observed between treatment groups. (**e**) Presence of exosome-sized, cup-shaped vesicles was verified by electron microscopy. FBS, fetal bovine serum. DC, differential centrifugation. TEI, Total Exosome Isolation (Life Technologies). All approximate protein masses are represented in kilodaltons (kDa).

Previously, reports suggested that highly pure vesicle preparations have particle-to-protein ratios in excess of ten billion^22^. To achieve such high purity ratios, samples are often prepared from cultures with low serum. To determine if highly purified preparations could be produced using the adapted PEG method, the cell culture procedure was modified to reduce serum contamination. Cells were cultured in complete medium that was changed to serum-free before vesicle harvests. PEG-based approaches were compared to sucrose-cushion purified vesicles, wherein samples were centrifuged into 30% sucrose and deuterium that was layered under the relatively less-dense culture media. The *sucrose cushion* method allows for very pure preparations, by maintaining the vesicles in a suspension layer of equal density to the vesicles^16^. All methods enriched for markers of exosomes relative to cell lysates, except with regard to detection of HSC70 (Fig. 3a; note: HSC70 was also highly abundant in cell lysates). Gels were stained to evaluate differences in proteins isolated (Fig. 3b). The *8% PEG* + *wash* method produced similar protein-banding patterns to the sucrose cushion. Each method produced highly pure preparations, exceeding purity ratios of 1.0 × 10^10^ (Fig. 3c). There was no statistical difference between the *8% PEG* + *wash* method and the sucrose cushion (*p* = 0.169). The *12% PEG* + *wash* preparations were less pure than the sucrose cushion samples (*p* = 0.005). No differences were observed in particle size (mean or mode) between groups (Fig. 3d). As the *8% PEG* + *wash* treatment efficiently enriched exosomes, comparable to the purity of the gold-standard differential centrifugation and sucrose cushion purification methods, we adopted this standard procedure and called it *ExtraPEG (Extracellular vesicle PEG-based precipitation protocol).*

To further verify purity and confirm the presence of vesicles, preparations were analyzed by electron microscopy. Small round particles with typical cup-shaped morphology were identified in all samples, verifying that exosomes were harvested (Fig. 3e)^15^.

### Mass spectrometry analysis of ExtraPEG enrichments identifies exosome proteins

**Figure 4:**
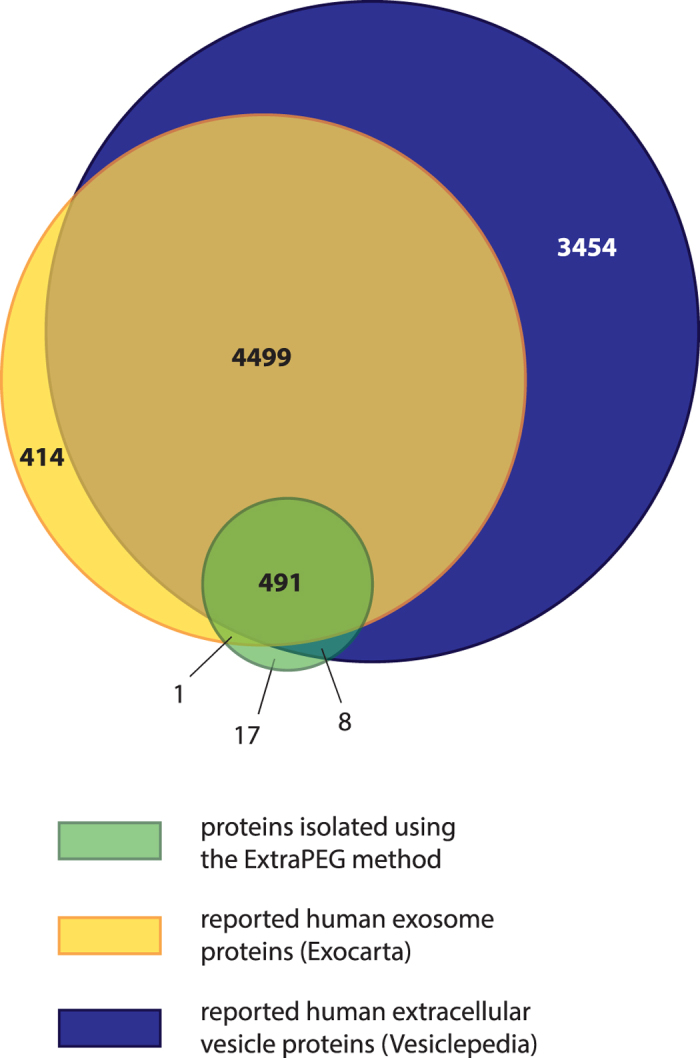
Extracellular vesicle proteins identified following ExtraPEG. Mass spectrometry analysis of ExtaPEG-harvested protein from HeLa cells identified 519 unique proteins. Approximately 97% (499 of 517) of ExtraPEG-identified proteins were found in the Vesiclepedia database of described human extracellular vesicle proteins. Additionally, 95% (492 of 517) of the proteins were previously characterized as exosome proteins. Of the 519 mass spectrometry identifications, 517 were used for this analysis as there were two sets of proteins for which a single gene name was used by the Exocarta and Vesiclepedia databases to represent multiple protein accession numbers (i.e.: HLA-A represented 1A68_HUMAN and 1A69_HUMAN; HLA-B represented 1B15_HUMAN and 1B55_HUMAN).

Though the PEG method produced pure vesicles enriched with exosomes, polymers like PEG can interfere with mass spectrometry. In order to test the practical utility of this method for downstream analyses, vesicles from cell culture were harvested and purified, and protein was analyzed by mass spectrometry. For this proteomics experiment, HeLa cells were cultured in serum-free media to minimize identification of non-vesicle protein, like bovine serum albumin, by mass spectrometry. The optimized ExtraPEG precipitation method (*8% PEG* + *wash*) was used to harvest extracellular vesicles, which were lysed, gel-purified, and in-gel digested. The resulting peptides were extracted and analyzed by liquid chromatography tandem mass spectrometry. Five hundred nineteen unique proteins were identified (Table S1). This list of identified proteins was compared to the Vesiclepedia database of all reported extracellular vesicle contents^23^. Four hundred ninety nine proteins were found in the Vesiclepedia search (about 97% of proteins identified), verifying the utility of the ExtraPEG method for use with proteomics experiments (Fig. 4). Of these proteins (common to both our study, and Vesiclepedia), 95% of the vesicle proteins were specifically classified as exosome proteins. Therefore, based on the known proteome of extracellular vesicles to date, these data suggest that the optimized PEG precipitation method successfully enriched extracellular vesicles including exosomes.

### ExtraPEG enriches exosomes from many biological fluids without inhibiting biological activity

As extracellular vesicles were successfully harvested from cell culture medium, we hypothesized that the described method would also work for harvesting from other biological fluids. To test this, we applied the protocol to mammalian plasma (mouse plasma; C57BL/6 and C3H genetic background), cerebral spinal fluid (CSF; human), urine (human) and saliva (human). The mode particle diameters measured from the resulting suspensions (~90 to 112 nm) were comparable to culture media enrichments using the very pure sucrose cushion method (~122 nm), as well as by the PEG method (~129 nm). Intriguingly, about ten times as many particles were harvested from the whole-organism isolates (urine, saliva, etc.) than were harvested from cell culture media (Table 1).

**Figure 5:**
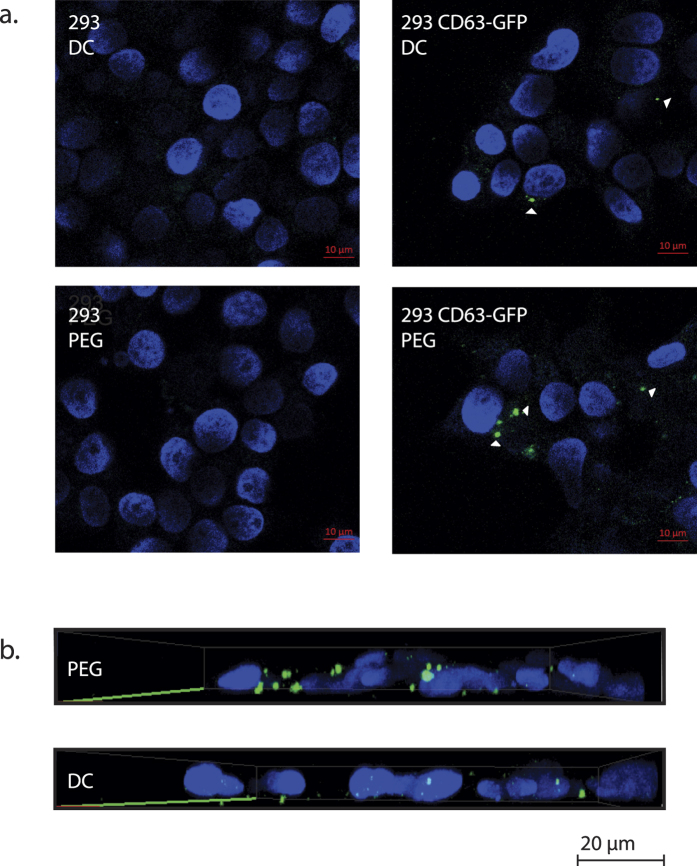
Efficient uptake of ExtraPEG isolated exosomes. (**a**) Confocal microscopy images of HEK293 cells. Cells were incubated for 24 hours with exosomes produced by HEK293 cells or HEK293 cells stably expressing CD63-GFP. Exosomes were harvested using ExtraPEG (PEG) or differential centrifugation (DC). (**b**) Three-dimensional image of combined z-stacks taken from cells exposed to PEG or DC-harvested CD63-GFP exosomes.

Polyethylene glycol minimally impacts the biological activity of vesicles. Yamamoto and Alberts (1970) found that with regard to viruses (ranging in size between 25 and 870 nanometers in length), 90 to 100% of the particles harvested remained infectious after being concentrated with PEG^24^. Since then, many studies have demonstrated the analogous uptake and biological activity of extracellular vesicles enriched with commercial products containing PEG^25^,^26^,^27^,^28^. However, to verify the ability of vesicles collected in this report to be taken up by recipient cells, we performed a vesicle transfer experiment. Here, exosomes containing green fluorescent protein (GFP) fused to the exosome protein CD63 were isolated from culture and incubated with a receiving cell line lacking GFP. Exosomes that were not carrying CD63-GFP were used as a negative control in these experiments. The CD63-GFP-containing exosomes were evaluated by confocal microscopy for their ability to be taken up by the recipient cells. The PEG-isolated vesicles were compared to fluorescent vesicles isolated using the gold standard differential centrifugation method. Vesicles enriched using PEG were taken up as well as, or better than those enriched using DC, a method that has also previously been shown to not inhibit biological activity of vesicles^5^ (Fig. 5).

### ExtraPEG isolation of exosomes preserves RNA cargo

**Figure 6:**
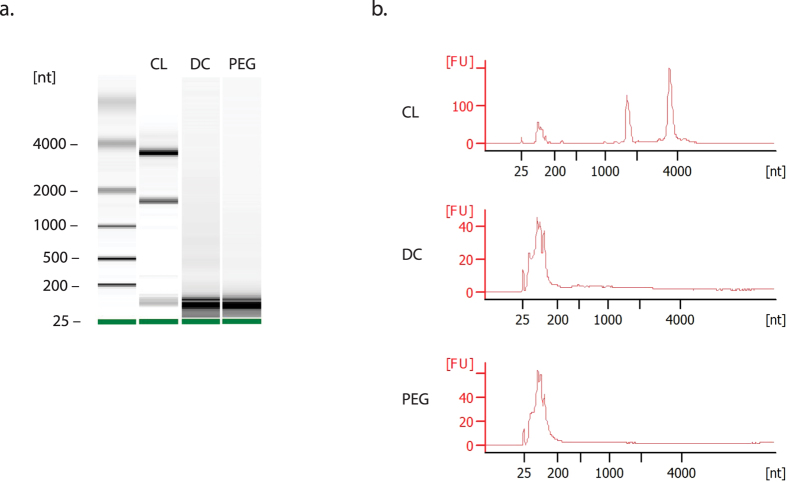
Extracellular vesicles harvested by ExtraPEG contain small RNAs. Exosome and cellular RNA were analyzed using the Agilent 2100 Bioanalyzer. (**a**) Gel-like image displaying RNA separated by size, for cell lysate (CL), differential centrifugation (DC), and the ExtraPEG (PEG) methods samples. (**b**) Electropherograms of RNA profiles from CL, DC, and PEG samples. [nt], nucleotide length. [FU], fluorescent units.

The ability of vesicles to associate with recipient cells suggested that effector proteins mediating binding and uptake were intact and functional. In addition to proteins, enzymes, and signaling molecules, exosomes contain messenger RNAs and microRNAs that are selectively packaged into the vesicles, and modify the activity of target cells^29^. In this way, small RNAs are also thought to be important effectors of intercellular communication^8^,^30^. It is critical therefore, that methods for isolating exosomes do so without affecting the integrity of RNA cargo. To assess the quality of RNA contained in vesicles enriched with PEG, isolated RNA was analyzed with an Agilent 2100 BioAnalyzer. The PEG and DC samples produced strong small RNA electropherogram peaks (at 25 to 200 nucleotides in length), characteristic of vesicle cargo. Notably, both were free of whole-cell ribosomal RNA (Fig. 6). High 28S:18S ribosomal RNA ratios in cell lysate (CL) samples verified that the RNA isolation technique was sound. These data further indicate that vesicles harvested by PEG are not adversely affected by the enrichment protocol, as compared to the gold standard (DC) method. The RNA can therefore be reliably used in downstream analyses.

## Discussion

In this study, we report that treating biological fluids with the ExtraPEG method (*8% PEG* + *wash*) yielded exosomes highly comparable to the gold standard differential centrifugation method, and superior to commercially available methods. This protocol was developed by adapting methods for harvesting viruses of similar morphological and chemical properties. Our first experiments tested the basic principle by treating cell culture media with varying concentrations of PEG and evaluating the resulting isolates for protein markers of exosomes. Six to eight percent PEG solutions produced the highest number of vesicles per contaminating protein (Fig. 1c–e). Four protein markers of exosomes were detected by western blot. Interestingly, HSC70 protein accumulated increasingly with greater PEG concentration, where the other exosome protein markers did not (Fig. 1e). It is possible therefore, that distinct subpopulations of HSC70-positive exosomes were present and were less readily precipitated by PEG. Alternatively, it could indicate that the presence of HSC70 was not specific to the extracellular vesicles in the cell culture medium. Numerous investigations suggest that secreted vesicles bear molecular markers specific to their progenitor cells; it is less clear how many subsets of exosomes exist and their distinct mechanisms of biogenesis. Subsequent characterization of extracellular vesicle populations will resolve this question.

Having proven the principle that exosomes, like virus particles, can be harvested with PEG, we next optimized the method and compared it to the gold standard differential centrifugation as well as commercial kits. Primary adaptations included washing particles with PBS after concentrating with PEG, and then re-concentrating by ultracentrifugation. This produced isolates highly comparable to DC, with less contaminating protein than commercial methods. By subsequently modifying the cell culture method (reducing serum content of the media), highly pure exosomes were recovered. We thus present ExtraPEG as a method to harvest vesicles that approximates, or exceeds, the gold standard in purity and quantity recovered, that is also easier and more cost-effective to execute. This method can be easily adapted for other purposes as well. Most simply, harvesting larger or smaller particles can be achieved by adding a filtration step, or applying greater or lesser centrifugation forces. As extracellular vesicles have received much attention for use as biomarkers of disease, ExtraPEG could also be modified for the clinical setting. Reports suggest that in some circumstances, as little as one hour incubation with PEG is sufficient for concentrating vesicles^31^. If having highly pure preparations is not required, it is conceivable that in concert with rapid diagnostic tests for exosome-biomarkers of disease, clinical samples could be evaluated in just over an hour without the need for subsequent purification steps.

Where some situations rely on the rapid and sensitive collection of vesicles, like perhaps in the clinic, others require highly pure isolates, such as those required for mass spectrometry analysis. After optimizing the ExtraPEG method, we therefore evaluated the potential for isolates to be further used for downstream analyses where purity is paramount. In preparation for proteomics analysis, vesicle protein was cleaned of PEG residue by running into a polyacrylamide gel. Others have reported various methods for removing PEG, as necessary, by size-exclusion or pressure filtration, column chromatography, cesium chloride sedimentation, dialysis, and potassium chloride precipitation^24^,^32^,^33^. Once purified and digested, protein was analyzed by mass spectrometry. About ninety five percent of proteins were previously identified as exosome proteins, including eighty-two of the one hundred most common proteins listed in the ExoCarta database, thus verifying the utility of this method for proteomics work. We also successfully isolated high quality RNA from exosomes, suggesting that the ExtraPEG procedure does not affect the stability of fragile small RNA species. As modifications to this protocol, such as centrifugation force and time, nano-filtration, and percent PEG used, can be applied for refining enrichments, this method serves as a practical basis for purifying exosomes and other extracellular vesicles for many purposes. As demonstrated, the method can also be used to enrich vesicles, from many biological fluids including mammalian plasma and cerebral spinal fluid. This simple salt solution containing PEG M_n_ 6000 costs less than 0.01 US dollars per milliliter of culture media used. This contrasts with commercial products that can cost close to six hundred times that amount. Because our versatile virus-adapted method produces comparable results, in terms of particle morphology, activity, and quantity, the price difference offers a substantial advantage over the commercial products, especially when considering the need to harvest large numbers of vesicles. Overall, ExtraPEG provides a simple and inexpensive means of harvesting extracellular vesicles from biological fluids that will be of use to many researchers in the field.

## Methods

### Cell culture

Human embryonic kidney 293T cells (HEK293T; ATCC^®^ CRL-11268™) were cultured in Dulbecco’s Modified Eagle’s Medium (DMEM; Lonza, 12-604Q). Medium was supplemented with either 0, or 10% fetal bovine serum (FBS; Seradigm, 1400–500) depleted of extracellular vesicles by centrifugation at 100,000 g (average relative centrifugal force) for 20 hr (4 °C). Cultures were incubated at 37 °C in an atmosphere supplemented with 5% carbon dioxide prior to collecting the media containing secreted extracellular vesicles. Cells were monitored daily for changes in growth rate, and in the proportion of live to dead cells to minimize production of other extracellular vesicle populations such as apoptotic bodies. In our hands, two million cells seeded into a 150 mm plate grew uniformly with consistent viability for up to four days (in 20 mL of complete growth media, with 10% vesicle-depleted FBS). Viability was determined by staining cells with AOPI (Nexcelom Bioscience, CS2-0106) and using an automated cell counter (Cellometer Vision, software version 2.1.4.2, Nexcelom Biosciences)^34^,^35^. For each experiment, harvested media from multiple flasks was pooled before distributing it into different sample groups.

For enrichment of vesicles for mass spectrophotometry analysis, HeLa cells (ATCC^®^ CCL-2™) were cultured as described above. After two days of growth, the complete medium was aspirated and cells were washed once with PBS warmed to 37 °C. To reduce contamination from serum proteins in the vesicle isolates, cells were cultured for a subsequent two days in serum-free medium.

### Differential centrifugation and sucrose cushion methods for enriching extracellular vesicles

Vesicles were harvested using a differential centrifugation method commonly used for enriching exosomes^16^, i.e.: conditioned media harvested from cell cultures was first centrifuged for five minutes at 500 g, to remove cells and larger debris. Media was then transferred to a new tube, and centrifuged at 2,000 g for ten minutes to remove cell debris and larger vesicles, such as apoptotic bodies. Large microvesicles were depleted by centrifugation at 10,000 g for 30 minutes. The media was then added to 35 mL polypropylene centrifuge tubes (Beckman Coulter Inc., #326823) in 35 mL aliquots, and centrifuged at 100,000 g (average relative centrifugal force) for 70 minutes using the SW-28 swing-bucket rotor in an Optima XL-100K ultracentrifuge (Beckman Coulter Inc.). Pelleted vesicles were suspended in 1 mL of PBS by gently vortexing, followed by agitating on an orbital shaker. The concentrated vesicle suspension was then centrifuged again in 1 mL polypropylene tubes (Beckman Coulter Inc., #347287) in an Optima MAX-E tabletop ultracentrifuge using a TLA120.2 rotor (Beckman Coulter inc.) to further purify the vesicles and facilitate re-suspension of the pellet in a small volume of PBS (50–100 μL). PBS was analyzed by NTA prior to suspension of vesicles and confirmed to contain particle numbers below the detection threshold of the instrument. The pellet was dissolved in particle free PBS by gentle vortexing followed by agitating on an orbital shaker for 15 to 30 minutes at room temperature until the solution was transparent and free of all aggregates. Samples were frozen at −80 °C until being further analyzed by nanoparticle tracking (see below) and protein quantification. All centrifugation steps were conducted at 4 °C. A subset of samples were processed as above, however, after centrifugation of the samples at 10,000 g, they were further centrifuged over a 30% sucrose cushion, as described by Thery *et al.*, to enhance the purity of the enrichment^16^. Briefly, four milliliters of sucrose solution was layered into 35 mL centrifugation tubes below a less-dense culture media layer. After centrifugation at 100,000 g for 75 min, 3.5 mL of the cushion (now containing the vesicles) was aspirated by piercing the tube and extracting with a 18-gauge syringe, carefully leaving any contaminants of greater, or lesser density. The 3.5 mL sucrose solution was then suspended in 31.5 mL PBS, and centrifuged a subsequent time at 100,000 g for 70 minutes to remove the sucrose. The purified pellet was re-suspended as described for the differential centrifugation procedure.

### Preparation of polyethylene glycol solution for precipitation of extracellular vesicles

Polyethylene glycol is commonly used to concentrate virus particles^21^. With this in mind, we modified and repurposed a well-reproduced method for virus enrichment in order to concentrate vesicles of comparable size and biochemical properties. Polyethylene glycol with M_n_ (number average molecular weight) of 6000 (Sigma, 81260) was combined with filtered water (18.3 Mega-ohm system; ELGA Purelab flex purification system) and sodium chloride (1 M) to make a two-fold concentrated (2x) stock solution. The 2x stock solution was added to an equal volume of substrate from which vesicles were to be harvested (see below). Concentrations between 5 and 15% were evaluated (2x stock solutions of 10 and 30% PEG, respectively) for the ability to recover particles between 40 and 1,000 nm, and vesicles containing exosome markers. Sodium chloride concentration was held constant at 0.5 M in all precipitation solutions.

The concentrated PEG stock solution was analyzed by nanoparticle tracking (see below) prior to use. Additionally, mock vesicle enrichments were conducted to ensure that no particles were generated by the reagents alone from the enrichment process. In both cases, particle numbers recovered were insignificant and below the reproducible threshold of instruments.

### Polyethylene glycol enrichment of extracellular vesicles

Vesicle-containing medium from cell culture was centrifuged at 500 g for five minutes followed by 2,000 g for thirty minutes at 4 °C to remove cellular debris and large apoptotic bodies. Once centrifuged, the media was added to an equal volume of a 2× PEG solution at 4 °C, to achieve a desired final PEG concentration (5–15%). After the 2× PEG solution was added, samples were mixed thoroughly by inversion, and incubated at 4 °C overnight (at least 12 hrs). The next day, samples were centrifuged in a tabletop centrifuge at maximum speed (Eppendorf, model 5810 R using an S-4-104 swing bucket rotor; 3,214 g) for 1 hour at 4 °C. Conical tubes were then decanted, and allowed to drain for five minutes, tapping occasionally to remove excess PEG. The resulting pellet was suspended in 50–500 μL of particle-free PBS (pH 7.4). Subsets of samples were then either stored at −80, or further purified by PEG-precipitation for a second time using a lower concentration of PEG, or by re-suspending in PBS and centrifuging at 100,000 g to wash and re-pellet the vesicles. For the former group of samples, the pellet resulting from the primary PEG treatment was diluted in 5 mL PBS. An equal volume of 2× PEG solution was added, this time to a lower final PEG concentration of 5%. The latter samples were suspended in 1 mL PBS and ultracentrifuged (100,000 g) for 70 minutes to wash the particles of contaminating protein and PEG. All samples were finally resuspended in particle-free PBS, by shaking at room temperature for up to 30 minutes.

### Enrichment of exosomes with polyethylene glycol-based commercial products

Two commercially available methods were used to enrich extracellular vesicles: the Total Exosome Isolation^TM^ (TEI) reagent from Life Technologies, and ExoQuick-TC™ from SBI. Vesicles were enriched according to the manufacturers’ instructions. For the TEI procedure, pooled media was aliquoted into 50 mL conical tubes and centrifuged at 2,000 g for 30 minutes. Supernatant was transferred to new tubes, and 0.5 volumes of TEI reagent was added and mixed thoroughly, before incubating overnight at 4 °C. The next day, samples were centrifuged at 10,000 g for 1 hour and the supernatant was then aspirated. The resulting pellet was suspended in PBS as described above. For the ExoQuick isolation, samples were drawn from the same pool of media (described above), and centrifuged in 50 mL conical tubes at 3,000 g for 15 minutes. The supernatant was added to a new tube, 1 mL of ExoQuick reagent was added for every 5 mL of culture media, and samples were mixed and incubated overnight at 4 °C (for at least 12 hours). Following this incubation, the suspension was centrifuged at 1,500 g for 30 minutes. Supernatant was then aspirated, followed by a brief centrifugation step (1,500 g for 5 min) to facilitate its further removal. The pellet was suspended as described above.

### Nanoparticle tracking analysis

To determine particle size and concentration, nanoparticle tracking analysis (NTA) was performed using the NanoSight^TM^ LM10-HS (Malvern Instruments) instrument configured with a blue (488 nm) laser and sCMOS camera. Samples (stored at −80 °C) were thawed and gently vortexed before further diluting in sterile, particle-free PBS at ratios of between 1:250 and 1:2,000 to achieve particle concentrations consistent with the optimal range of analysis with the software (between 2 × 10^8 and 1.8 × 10^9 particles per mL). PBS was tracked before each experiment to ensure that it was particle-free. Samples were manually mixed three times by aspirating and expelling the diluted particle suspension using a 1 mL syringe. Each sample was then injected into the laser chamber. Three 60-second recordings were performed for each sample and technical replicates were averaged. Camera shutter speed was fixed at 30.00 milliseconds. The NTA 3.0 software was used to measure the mode and mean size, and the concentration of particles per 1 mL solution. Camera level was set to 13, and detection threshold was maintained at 3 to ensure accurate and consistent detection of small particles. The laser chamber was cleaned thoroughly between each sample reading with particle-free water and 70% ethanol.

### Transmission electron microscopy

Following ExtraPEG, DC and sucrose cushion methods, exosome-enriched isolates were resuspended in 50–100 μL of sterile filtered PBS for electron microscopy imaging. Sample preparation was done according to Lasser *et al.*^36^.

### Protein quantification, western blots, and antibodies

Exosomes were lysed by adding an equal volume of a urea-containing strong lysis buffer to the vesicles suspended in PBS^37^. This lysate was agitated at room temperature for 15 minutes on a mechanical shaker. The concentration of protein was assessed using the EZQ™ Protein Quantitation Kit (Life Technologies, R-33200), and 1.7–8.0 μg of protein was loaded into 4–20% sodium dodecyl sulfate polyacrylamide gels (Lonza, #59111). For western blotting, proteins were transferred to a nitrocellulose membrane (GE Healthcare, #10600002). The membranes were blocked with 5% (weight/volume) nonfat dry milk powder suspended in a standard tris-buffered saline solution with tween 20 (TBST), either overnight at 4 °C or for one hour at room temperature. Membranes were then probed for exosome proteins using up to four antibodies: TSG101 (Santa Cruz Biotechnology; SC-7964), HSC70 (Santa Cruz Biotechnology; SC-7298), CD63 (Santa Cruz Biotechnology; SC-15363), and ALIX (Santa Cruz Biotechnology; SC-49268). These primary antibodies were subsequently probed with appropriate secondary antibodies conjugated to horseradish peroxidase. Enhanced chemiluminescent (ECL) HRP substrate was added for picogram (Thermo Scientific, #1856136) or femtogram (Amresco, 1B1583) protein detection thresholds. Chemiluminescence was detected using the LAS4000 luminescent image analyzer and software Version 8.1 of Image Quant-TL (GE Healthcare Life Sciences). Cropped blot images were compiled from either trimmed segments of a single nitrocellulose membrane that were probed for different proteins, or from different blots where protein molecular weights overlapped. In the latter case, blots to be combined were run under identical conditions using aliquots of the same sample stocks.

### Mass spectrometry analysis

HeLa cell vesicles were harvested using the ExtraPEG method (*8% PEG* + *wash*), and lysed. Recovered protein was quantified using the EZQ quantification kit, and 6.5 μg of protein was purified by tris-glycine sodium dodecyl sulfate polyacrylamide gel electrophoresis (SDS-PAGE). Protein was run into the 4–20% polyacrylamide gel slowly, at 50 volts for approximately 25 min. The gel was fixed and incubated in a Coomassie Brilliant Blue G-250 based stain^38^, imaged, and regions containing protein (protein was run approximately 1 cm into the gel lanes) were excised. The gel pieces containing protein were subdivided into 1 mm^3^ pieces before placing into microcentrifuge tubes (1 tube per sample). They were then washed with 1 mL of HPLC grade water, and destained by shaking in 500 μL of 50 mM ammonium bicarbonate and 50% methanol for ten minutes (repeated until gel cubes appeared clear). The cleared gel pieces were subsequently washed with 1.2 mL of 50 mM ammonium bicarbonate in 50% acetonitrile for five minutes with shaking, followed by dehydration in 100% acetonitrile for one minute. Samples were then further desiccated by vacuum centrifugation for five minutes. The dehydrated gel pieces were re-suspended in 400 μL of a reducing solution containing 25 mM dithiothreitol in 50 mM ammonium bicarbonate and incubated at 56 °C for 20 min with gentle shaking. Reduction was followed by alkylation of samples by removing reducing solution and adding the same volume of 55 mM iodoacetamide in 50 mM ammonium bicarbonate. Samples were incubated in the dark for 20 min. Iodoacetamide was removed, and samples were washed with 1 mL of ultra-pure water by vortexing. After two more washes, water was removed and gel pieces were slowly dehydrated by adding 1,000 μL of 50 mM ammonium bicarbonate in 50% acetonitrile, followed by a subsequent incubation in an equal volume of 100% acetonitrile, and desiccation by vacuum centrifugation as before. Finally, gel pieces were cooled on ice, and rehydrated with Trypsin Gold protein digest enzyme (Promega, V5280) suspended in ProteaseMAX™ Surfactant Trypsin Enhancer (Promega, V2071) according to the manufacturer’s recommendations. The digestion reaction solution with extracted peptides was aspirated and placed into new tubes. Peptides were snap-frozen on dry ice and dried completely in the vacuum centrifuge at room temperature. Samples were then submitted to the Translational Science Laboratory at Florida State University for tandem mass spectrometry analysis. Liquid chromatography tandem mass spectrometry (LC-MS/MS) analysis was done using an externally calibrated Thermo LTQ Orbitrap Velos nLC-ESI-LIT-Orbitrap (high-resolution electrospray tandem mass spectrometer) with parameters set as previously described^39^. Raw data files produced were loaded into Proteome Discoverer™ Software (Thermo Fisher Scientific Inc.) and database searches were conducted for each technical replicate with Sequest HT and Mascot (version 2.4.0) using the UniProt Knowledge Base Swiss-Prot Homo Sapiens database. This reference database was appended with sequences of common contaminants of proteomics studies to minimize false positive identifications. Contaminant sequences in the common Repository of Adventitious Proteins (cRAP) were downloaded from the Global Proteome Machine website^40^. Files (.msf) were then loaded into Scaffold Proteome Software (Scaffold PTM 2.2.0) and data was analyzed using a false protein discovery rate of 1.0% with a minimum of two peptides required for identification. The mass spectrometry proteomics data have been deposited to the ProteomeXchange Consortium^41^ via the PRIDE partner repository with the dataset identifier PXD003209.

### Exosome enrichment from bodily fluids

To confirm the utility of ExtraPEG in isolating vesicles from biological fluids, de-identified human cerebral spinal fluid, urine, and saliva were purchased from BioreclamationIVT (Hicksville, New York). BioreclamationsIVT declares all samples were collected under IRB approved protocols. Mouse plasma was generously donated by Dr. James Olcese (Florida State University). All mouse samples were obtained under a Florida State University Animal Care and Use Committee approved protocol. Vesicles were enriched from biological samples using the ExtraPEG method and quantified using nanoparticle tracking analysis.

### Stable cell line generation

HEK293 cells stably expressing CD63-GFP were generated by lentivirus transduction and puromycin selection using pCT-CD63-GFP (Systems Biosciences Inc.). Medium containing lentivirus particles was harvested from HEK293-T cells transfected with pCT-CD63-GFP and packaging plasmids pMD2.G (Addgene, #12259), pRSV-Rev (Addgene #12253), and psPAX2 (Addgene #12260, kind gifts from Dr. Didier Trono^42^) at 48 hr post-transfection. Medium was then centrifuged for 10 min at 1,000 g and filtered through a 0.45 μM filter. HEK293 cells were incubated with the resulting lentivirus-containing medium and a 10 μg/mL final concentration of Polybrene (Sigma, #9268). Following 24 hr of incubation, the medium was replaced with complete DMEM for 24 hr and then puromycin was added at a final concentration of 2 μg/mL. Cells were subcultured for three weeks under puromycin selection to eliminate non-transduced cells.

### Exosome uptake assay

One hundred fifty millimeter dishes of HEK293 or HEK293-expressing CD63-GFP cells were grown in medium containing 5% exosome-depleted FBS for 48 hr. The conditioned medium was harvested using the ExtraPEG or DC method described above, and the resulting exosome pellets were re-suspended in 1 mL of DMEM containing 5% exosome-depleted FBS and incubated with HEK293 cells grown on glass cover slips in 35 mm dishes. Twenty-four hours post incubation with exosome-containing medium the cells were wasted twice with PBS and fixed with 4% paraformaldehyde for 10 min at room temperature. The fixed cells were washed three times with PBS, stained with a 1:10,000 dilution of DAPI (4′,6-diamidino-2-phenylindole; 1 mg/mL stock, Thermo Fisher Scientific Inc.) in PBS, and mounted on glass slides with a drop of mounting media (4% propyl gallate and 90% glycerol in PBS). Confocal images were obtained with a 63X objective on a Zeiss LSM 880 microscope and processed using the Zen 2 (Blue edition, V2.0.0.0; Zeiss) software package.

### Quantitation of total exosome RNA

Exosomes from HEK293T cells were enriched by differential centrifugation and ExtraPEG method (*8% PEG* + *wash*). RNA was extracted using Invitrogen’s Total Exosome RNA and Protein Isolation kit (#4478545) according to the manufacturer’s recommendations: exosome pellets were re-suspended in phosphate buffered saline, denatured, and RNA was purified by acid-phenol/chloroform extraction. As a control, RNA from HEK293T cell lysate was similarly purified. Concentrations of 2,500 picograms per microliter were loaded onto an Agilent 6000 pico chip and total RNA was quantitated using an Agilent 2100 Bioanalyzer. Gel-like images and electropherograms were generated by Agilent 2100 Expert Software (version B.02.08.SI648).

### Statistical analysis

Statistical analyses were performed using Stata 10 data analysis and statistical software (Stata Corp LP). All sample populations evaluated were compared with Bartlett’s test for equal variance. Differences between two groups were assessed using Student’s t-test (two-tailed) or the Wilcoxon rank-sum test where appropriate. Comparisons of three or more groups were conducted using a one-way ANOVA, with the Bonferroni multiple comparison procedure. Significance of linear regression models and population comparisons was set to α = 0.05. Proteomics analysis was conduction using Vesiclepedia (http://www.microvesicles.org/) and Exocarta (http://www.exocarta.org/) databases of extracellular vesicle and exosome contents, respectively^23^,^43^. Only human proteins from these databases were used for comparison to the proteins identified in this study. Venn diagrams were generated with BioVenn^44^ and Adobe Illustrator CS6 (Adobe Systems Inc.).

## Additional Information

**How to cite this article**: Rider, M. A. *et al.* ExtraPEG: A Polyethylene Glycol-Based Method for the Enrichment of Extracellular Vesicles. *Sci. Rep.* **6**, 23978; doi: 10.1038/srep23978 (2016).

## Supplementary Material

Supplementary Information

## Figures and Tables

**Table 1:** ExtraPEG enriches extracellular vesicles from biological fluids.

Body fluid	Mode particlesize (nm)	Mean particlesize (nm)	Particles per μL ofbody fluid (×10^8^)
CSF	111.6 ± 14.9	135.4 ± 9.4	0.83 ± 0.39
plasma	90.2 ± 3.6	120.6 ± 3.3	1.56 ± 0.23
saliva	107.9 ± 6.4	161.7 ± 8.1	2.41 ± 0.53
urine	110.2 ± 9.4	163.6 ± 18.0	2.42 ± 0.48

Extracellular vesicles were harvested from mammalian body fluids using ExtraPEG. The size and quantity of particles was measured by NTA. Mean ± SEM.
